# Psychometric properties of the osteoporosis assessment questionnaire (OPAQ) 2.0: results from the multiple outcomes of raloxifene evaluation (MORE) study

**DOI:** 10.1186/1471-2474-15-374

**Published:** 2014-11-17

**Authors:** Wei Shen, Russel Burge, April N Naegeli, Jeremy Shih, Jahangir Alam, Deborah T Gold, Stuart Silverman

**Affiliations:** Eli Lilly and Company, Indianapolis, IN USA; inVentiv Clinical Solutions, Baltimore, MD USA; Duke University Medical Center, Durham, NC USA; Cedars-Sinai/UCLA, Beverly Hills, CA USA

**Keywords:** Health-related quality of life, Osteoporosis, Vertebral fracture, Psychometric properties

## Abstract

**Background:**

We explored psychometric properties of the Osteoporosis Assessment Questionnaire 2.0 in terms of reliability, validity, and responsiveness with generic, clinical, demographic, and preference-based data collected from a population of postmenopausal women with osteoporosis.

**Methods:**

The Multiple Outcomes of Raloxifene Evaluation study was a randomized, placebo-controlled, multinational clinical trial evaluating efficacy and safety of raloxifene. The Osteoporosis Assessment Questionnaire 2.0, a generic quality of life measure (Nottingham Health Profile), and a preference-based measure (Health Utilities Index) were administered at baseline and annually. Psychometric properties of the 14 Osteoporosis Assessment Questionnaire 2.0 domains were evaluated by standard statistical techniques.

**Results:**

This study included a subset of 1477 women from the Multiple Outcomes of Raloxifene Evaluation study population completing the questionnaires. Mean (standard deviation) age was 68.4 (6.8) years. Prevalent vertebral fractures were found in 70% (n =1038) of women. Internal consistency was >0.7 in 9 Osteoporosis Assessment Questionnaire 2.0 domains. Correlations were moderate and significant for similar Osteoporosis Assessment Questionnaire 2.0 domain scores, Nottingham Health Profile domains, and Health Utilities Index scores. All but 2 Osteoporosis Assessment Questionnaire 2.0 domains distinguished between patients with or without prevalent vertebral fractures and detected worsening with increased number of vertebral fractures. Women with ≥1 incident vertebral fracture generally had a greater worsening in Osteoporosis Assessment Questionnaire 2.0 scores (excluding social activity and support of family and friends) from baseline to study endpoint compared with women without incident vertebral fractures.

**Conclusions:**

Most domains in the Osteoporosis Assessment Questionnaire 2.0 demonstrated robust psychometric properties; however, several domains not showing these criteria may need to be reassessed and removed for a potentially shorter and validated version of the Osteoporosis Assessment Questionnaire.

**Electronic supplementary material:**

The online version of this article (doi:10.1186/1471-2474-15-374) contains supplementary material, which is available to authorized users.

## Background

Osteoporosis is a chronic disease in which bone mineral density (BMD) is reduced and structural deterioration of the bone tissue occurs, which leads to bone weakness and an increased susceptibility to fractures [1, 2]. In postmenopausal women, osteoporosis is the major underlying cause of fractures, which often occur in the hip, spine, and wrist [1–4]. Health-related quality of life (HRQoL) is a multidimensional concept that defines a person’s health status in specific dimensions including physical, social, emotional, and functional well-being [5]. Osteoporosis also can impact multiple dimensions of HRQoL, including: anxiety and depression, reduced self-image, limitations in the ability to work and enjoy leisure activities, acute or chronic pain, difficulties in performing the activities of daily life, loss of independence, and changes in relationships with family and friends [3, 6]. In women with established postmenopausal osteoporosis, vertebral fractures may result in back pain, physical functioning limitations, and psychosocial impairment [7, 8].

Assessment of HRQoL in women with osteoporosis remains an important objective, especially among those women with severe osteoporosis (especially those with fracture). Despite recent progress in the treatment of osteoporosis (e.g. more treatment options are available), there has been limited progress in the development of osteoporosis-specific quality of life instruments over the last decade. The HRQoL among patients with osteoporosis—as measured by disease-targeted instruments such as the Osteoporosis Assessment Questionnaire (OPAQ)—decreases following incident clinical fracture [6]. The OPAQ is an 81-item, validated instrument that was developed with patients and healthcare professionals which shows adequate psychometric properties and appropriateness for use during clinical trials [9–11]. Some items from the OPAQ that did not discriminate between patients with and without prevalent vertebral fracture in the Sanofi tiludronate trial were subsequently eliminated to create a short-form, 67-item OPAQ instrument version 2.0 (OPAQ 2.0) [12]. The recall period was also changed from 4 to 2 weeks to improve accuracy of recall. The OPAQ 2.0 is a disease-targeted, patient-reported measure of HRQoL in patients with osteoporosis. The questionnaire was 1 of 2 disease-targeted instruments administered to measure HRQoL in the Multiple Outcomes of Raloxifene Evaluation (MORE) study.

The MORE study was the first large interventional trial in osteoporosis to perform prospective HRQoL assessments over a 3-year period [6, 13–15]. While the primary objective of the MORE study was to examine the long-term effects of raloxifene on the skeleton in postmenopausal women with osteoporosis, the secondary objective was to compare treatment-related changes in HRQoL. The MORE study design and results have been reported elsewhere; in summary, both prevalent and incident vertebral fractures were associated with decreases in HRQoL, and increasing numbers of prevalent vertebral fractures were associated with progressive decreases in HRQoL [6, 14]. According to the study results, the HRQoL effect of vertebral fracture depends on the number and location of fractures [6, 10, 14].

The validity and clinical relevance of HRQoL instruments have come under increased scrutiny since the 2005 European Medical Agency and 2009 United States Food and Drug Administration guidelines related to the use of patient-reported outcomes (PRO) in clinical medical product development [16, 17]. These guidelines clearly specify a need to develop and confirm the suitability of HRQoL instruments in the patient population for which the therapy will be indicated in order to support the validity of evaluation. The HRQoL data from the MORE trial remain a robust and rich source of HRQoL information in osteoporosis clinical trials. The MORE trial participants were generally in the early stage of osteoporosis, although MORE also included a sizable number of patients with severe osteoporosis who were administered questionnaires including the OPAQ 2.0. Therefore, the main objective of this study was to explore the psychometric properties of the OPAQ in terms of reliability, construct validity, and responsiveness by using PRO, clinical (e.g. fracture), demographic (e.g. age), and preference-based data collected from women in the MORE study.

## Methods

### Study population

**Figure 1:**
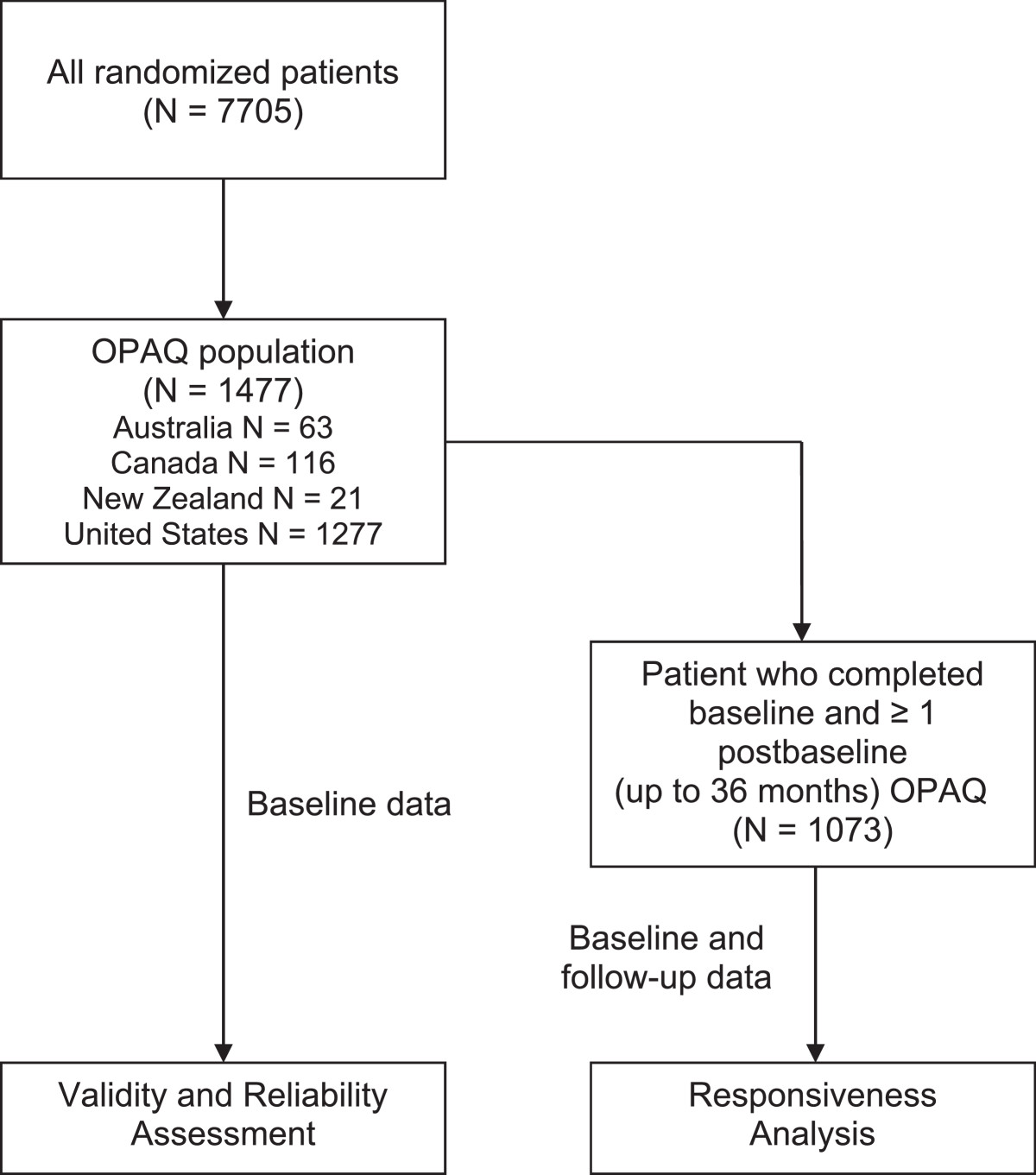
**Population of women included in the validity and reliability assessment and the responsiveness analysis.** Abbreviation: N = number; OPAQ = Osteoporosis Assessment Questionnaire.

This was a post hoc retrospective analysis that used data from the MORE study. The MORE study was a randomized, placebo-controlled, multinational clinical trial designed to evaluate the efficacy and safety of raloxifene. Participants in the MORE study included 7705 postmenopausal women, aged ≤80 years at 180 centers in 25 countries. Women, who had osteoporosis, as defined by low BMD (T-score ≤ −2.5 standard deviations below the young adult peak mean BMD) or radiographically apparent vertebral by fractures, were enrolled into 2 study groups and then randomly assigned to 1 of 3 treatment groups. Study group 1 included those whose femoral neck or lumbar spine BMD T-score was below −2.5. Study group 2 included women who had low BMD and ≥1 moderate or severe vertebral fracture; low BMD and 2 mild vertebral fractures; or at least 2 moderate vertebral fractures, regardless of BMD [13]. The MORE study protocol was approved by the human studies review board at each center, and informed consent was obtained. The MORE clinical study was conducted according to the ethical principles stated in the latest version of the Declaration of Helsinki, the applicable guidelines for good clinical practices, or the applicable laws and regulations of the countries where the study was conducted, whichever provided the greater protection of the individual. For the current study on validity and reliability assessment, the analyses included all 1477 patients who completed the OPAQ 2.0 at baseline, and for responsiveness analyses, patients who completed baseline and ≥1 annual post-baseline measure (up to 36 months) were included (Figure 1).

### Clinical and health-related quality of life measurements

Participants underwent spine radiography at baseline, 24 months, and 36 months. Women were seen every 6 months over the 3 years of the MORE study. All vertebral fractures were confirmed by review of spine radiographs, and patients were informed of the results. Incident vertebral fractures were assessed at scheduled yearly follow-up visits or at unscheduled visits, according to reported symptoms suggestive of a fracture, but fractures were always confirmed by radiographic evidence. Nonvertebral fractures were determined by direct questioning, every 6 months at each clinic visit. Spine and femoral neck BMD were measured at baseline and annually by dual-energy x-ray absorptiometry. Nonvertebral fractures (i.e. humerus, wrist, hip, patella, tibia/fibula, ankle, metatarsal, rib/sternum, clavicle, scapula, sacrum, and pelvis) were assessed by self-report. Demographic and patient characteristics were collected at baseline. The OPAQ 2.0 (osteoporosis-specific HRQoL questionnaire) was administered at baseline and annually, alongside a generic measure of quality of life (Nottingham Health Profile [NHP]) and a preference-based measure (Health Utilities Index [HUI]).

### OPAQ version 2.0

The OPAQ 2.0 is a validated, self-administered HRQoL instrument that consists of 67 questions (Additional file 1). It contains 6 questions about general health, overall HRQoL, and current living situation; 12 questions about importance of daily activity; and 49 questions in 14 osteoporosis-targeted domains, which yielded 4 composite dimensions when combined through factor analyses (Additional file 2): physical function, emotional status, symptoms, and social interaction. The physical function dimension includes 6 domains: walking/bending, standing/sitting, dressing/reaching, household/self-care, transfers, and usual work. The emotional status dimension includes 4 domains: fear of falls, level of tension, body image, and independence. The symptoms dimension includes 2 domains: back pain and fatigue. The social interaction dimension includes 2 domains: social activity and support of family and friends. Measurement properties of the 4 composite dimensions have been reported previously [6].

The developer’s scoring algorithms for the OPAQ 2.0 are described below [9, 10]).

1. Selecting individual questions: A total of 48 questions (Questions 7 through 55) are used to create 14 OPAQ domains. All 48 questions take on values 1, 2, 3, 4, or 5.
2. Recoding: Because the OPAQ 2.0 is scored such that a high value indicates better health status, it was necessary to recode several items before calculating domain and dimension scores to avoid systematic response biases. Thus, 17 of the 48 questions were reverse-scored so that a response of 5 indicates the best possible quality of life, and 1 indicates the worst quality of life. For the remaining items, 1 indicated the best possible quality of life and 5 indicated the worst quality of life.
3. Imputing missing data: A missing value was imputed only if at least one-half of the questions, within the same domain, were answered. If so, the missing value was replaced by the average of the nonmissing values in the same scale.
4. Forming a domain score: Values within the same domain were added to form a domain score. If more than one-half of the question responses were missing, the domain score was set to missing.
5. Transformation of domain scores: All domain scores were transformed to a range of 0 to 100, with 100 indicating the best HRQoL.

The NHP and HUI were scored according to the user manuals. The NHP domain scores range from 0 to 100, with lower scores indicating lower level of distress (or better quality of life) [18]. The HUI scores range from 0 to 1, with higher scores indicating better health utility [19]. Both NHP and HUI have previously been validated [18, 19].

### Statistical analyses

Psychometric properties of the OPAQ 2.0 domains were evaluated by standard statistical techniques. Internal consistency reliability was assessed by Cronbach's alpha (>0.7 was considered acceptable) [20].

Construct validity was tested in 2 ways. First, convergent validity between OPAQ 2.0 domains and corresponding NHP domains and HUI scores were examined by use of Pearson’s correlation coefficient. Correlations, which demonstrate validity, typically range from 0.30 to 0.80 [21]. We hypothesized that the OPAQ 2.0 domain scores would be significantly and meaningfully associated with corresponding NHP domains and HUI scores (e.g. OPAQ 2.0 walking/bending vs. NHP mobility, and OPAQ 2.0 back pain vs. NHP pain). By use of a criterion suggested by Guilford and Fruchter [21], a significant correlation coefficient ≤ -0.30 or ≥0.30 [absolute value], between the OPAQ 2.0 domain and corresponding NHP domain and HUI score was considered meaningful (i.e. supportive of the construct validity of the OPAQ 2.0). Second, discriminant validity was assessed by comparing OPAQ 2.0 domain scores between several known groups by using analysis of covariance with country of origin, age, body mass index (BMI), years since menopause, smoking status (yes vs. no), alcohol consumption (yes vs. no), and number of preexisting conditions included in the model:

1. Presence of prevalent vertebral fracture (0 vs. ≥1, 0–1 vs. ≥2, and 0 vs. ≥2) [6, 14].
2. Presence of prevalent osteoporotic nonvertebral fracture (0 vs. ≥1 and 0–1 vs. >1) [22]. Nonvertebral fractures included 12 locations: humerus, wrist, hip, patella, tibia/fibula, ankle, metatarsal, rib/sternum, clavicle, scapula, sacrum, and pelvis.
3. Trend analysis for age (<65, 65 ≤ age ≤70, and >70) [23].
4. Baseline femoral neck BMD T-scores (≥ −2.5 vs. < −2.5) [24].

In each of the known groups above, we hypothesized that OPAQ 2.0 domain scores would be lower for the former group when compared to those of the latter group. An additional analysis was performed, by using multiple linear regression models, to examine the differences in OPAQ 2.0 domain scores with an increasing number of prevalent vertebral fractures.

Mean changes in OPAQ 2.0 domains from baseline to endpoint, were compared between patients with and without incident vertebral fractures. Incident fracture is a meaningful clinical endpoint for patients with established osteoporosis, and it was the primary endpoint in the MORE study. It was hypothesized that HRQoL would decrease among patients with incident vertebral fractures; therefore, a HRQoL instrument with good responsiveness would show differences between those patients who have incident fractures versus those who do not. Responsiveness (i.e. sensitivity to clinical change) was assessed by comparing OPAQ 2.0 score change from baseline to study endpoint between patients with and without incident vertebral fractures, by using ANCOVA adjusted for country of origin.

## Results

The demographic and clinical characteristics of participants are shown in Table 1. The 1477 women were predominantly white (96%) with a mean (standard deviation) age of 68.4 (6.8) years. Prevalent vertebral fractures were found in 70% (n =1038) of women; the mean (standard deviation) number of prevalent vertebral fractures (40 days before baseline) was 1.32 (1.38).

**Table 1:** **Demographic and clinical characteristics**

Parameter	Value
**Sample size, N**	1477
**Age, years, mean (SD)**	68.42 (6.81)
**Body mass index, kg/cm** ^**2**^ **, mean (SD)**	25.73 (4.34)
**Years postmenopause, mean (SD)**	20.94 (8.65)
**Racial origin, white, n (%)**	1417 (96%)
**Prevalent vertebral fracture** ^**a**^ **, n (%)**	1038 (70%)
**Femoral neck BMD T-scores, mean (SD), [min, max]**	−2.39 (0.57), [−4.5, 0.03]
**Country of origin, n (%)**	
Australia	63 (4.3%)
Canada	116 (7.9%)
New Zealand	21 (1.4%)
United States	1277 (86%)
**Smoking status** ^**a**^ **(yes vs. no, n)**	180 vs. 1271
**Alcohol consumption (yes vs. no, n)**	306 vs. 1171
**Number of prevalent vertebral fracture** ^**a**^	
Mean (SD)	1.32 (1.38)
>Zero vs. zero	1038 vs. 439
**Number of osteoporotic nonvertebral fracture**	
Mean (SD)	0.98 (1.24)
>Zero vs. zero	797 vs. 680
**Baseline lumbar spine BMD, mean (SD), [min, max]**	−2.51 (1.20), [−6.0, 3.0]
**Family history of osteoporosis (yes vs. no/unknown)**	469 vs. 1008
**History of hysterectomy (yes vs. no, n)**	411 vs. 1066
**Marital status** ^**a**^ **(married vs. other, n)**	823 vs. 648
**Years of education, mean (SD)**	13.81 (3.03)
**Number of preexisting conditions, mean (SD)**	10.37 (5.92)

Abbreviations: BMD = bone mineral density; min = minimum; max = maximum; n/N = number; SD = standard deviation; vs. = versus.

^a^Twenty-six subjects had no record of smoking status; six subjects had no record of marital status; three subjects had no record regarding number of prevalent vertebral fracture.

Table 2 summarizes baseline distribution of scores and Cronbach’s alpha for each OPAQ 2.0 domain. The internal consistency of 9 domains were acceptable (Cronbach’s alphas >0.7) and 4 domains had Cronbach’s alphas between 0.6 and 0.7 (dressing/reaching [0.68], household/self-care [0.61], fatigue [0.68], and social activity [0.66]).

**Table 2:** **Baseline distribution and internal consistency of OPAQ scales**

OPAQ Scales	N	Mean	SD	Min	Max	Cronbach’s Alpha
Walking/bending	1471	84.24	18.0	4	100	0.82
Standing/sitting	1472	78.71	22.2	0	100	0.73
Dressing/reaching	1471	92.05	16.4	0	100	0.68
Household/self-care	1469	91.58	14.9	13	100	0.61
Transfers	1468	90.18	19.0	0	100	0.88
Usual work	1471	90.52	18.1	0	100	N/A
Fear of falls	1473	71.86	20.8	0	100	0.82
Level of tension	1469	66.95	18.0	10	100	0.90
Body image	1468	64.56	25.9	0	100	0.76
Independence	1469	82.75	17.8	0	100	0.71
Back pain	1475	71.72	24.6	0	100	0.86
Fatigue	1472	61.82	19.6	0	100	0.68
Social activity	1470	40.17	19.4	0	100	0.66
Support, family and friends	1469	85.06	20.8	0	100	0.78

Abbreviations: N/A = not applicable, the scale of Usual Work only has one OPAQ item 44; OPAQ = Osteoporosis Assessment Questionnaire; SD = standard deviation.

As expected, correlations were moderate and significant for similar OPAQ 2.0 domains and NHP domains and HUI scores. Table 3 provides a comprehensive summary of correlations between OPAQ 2.0 and the other 2 instruments (NHP and HUI). All correlations between OPAQ 2.0 and NHP were negative, which indicates that better HRQoL measured by OPAQ 2.0 was correlated with lower levels of distress measured by NHP. Correlations for OPAQ 2.0 walking/bending versus NHP physical mobility (r = −0.744) and OPAQ 2.0 back pain versus NHP pain (r = −0.669) were substantial. Correlations between OPAQ 2.0 and HUI were positive, which indicates better HRQoL measured by OPAQ 2.0 was correlated with high utility score measured by HUI. Correlations between all NHP domains and the OPAQ 2.0 domain for body image were < |0.35| and were statistically significant (p <0.0001). Correlations between the NHP domains and OPAQ 2.0 domains social activity (NHP physical mobility r = −0.094, NHP pain r = −0.075, NHP sleep r = −0.089; p <0.05) and support of family and friends (NHP physical mobility r = −0.096, NHP pain r = −0.093; p <0.05) were < |0.35| and were statistically significant. Similarly, correlations between HUI scores and OPAQ 2.0 domains were <0.3 for body image (r =0.266, p <0.0001), social activity (r =0.115, p <0.05), and support of family and friends (r =0.093, p <0.05).

**Table 3:** **Convergent validity: association between OPAQ, HUI, and NHP scales**

OPAQ Scales	NHP Emotional Reaction	NHP Energy	NHP Physical Mobility	NHP Pain	NHP Sleep	NHP Social Interaction	HUI
Walking/bending	−0.318	−0.567	−0.744	−0.609	−0.321	−0.226	0.525
Standing/sitting	−0.293	−0.524	−0.710	−0.667	−0.308	−0.196	0.491
Dressing/reaching	−0.211	−0.369	−0.567	−0.435	−0.213	−0.166	0.326
Household/self-care	−0.189	−0.380	−0.569	−0.441	−0.194	−0.171	0.336
Transfers	−0.254	−0.453	−0.679	−0.598	−0.279	−0.178	0.417
Usual work	−0.294	−0.509	−0.554	−0.468	−0.287	−0.230	0.401
Fear of falls	−0.352	−0.462	−0.607	−0.459	−0.304	−0.209	0.456
Level of tension	−0.587	−0.434	−0.312	−0.279	−0.378	−0.349	0.341
Body image	−0.249	−0.307	−0.319	−0.290	−0.227	−0.153	0.266
Independence	−0.304	−0.453	−0.571	−0.444	−0.289	−0.230	0.403
Back pain	−0.294	−0.493	−0.648	−0.669	−0.335	−0.173	0.496
Fatigue	−0.407	−0.607	−0.476	−0.455	−0.481	−0.246	0.443
Social activity	−0.156	−0.148	−0.094*	−0.075*	−0.089*	−0.214	0.115*
Support, family and friends	−0.293	−0.171	−0.096*	−0.093*	−0.105	−0.305	0.093*

Abbreviations: HUI = Health Utilities Index; NHP = Nottingham Health Profile; OPAQ = Osteoporosis Assessment Questionnaire.

Values shown are Pearson correlation coefficients.

*p <0.05; All other p-values <0.0001.

All but 2 OPAQ 2.0 domains (level of tension and support of family and friends) were able to discriminate between patients with or without prevalent vertebral fractures and were associated with a worsening trend with increased number of vertebral fractures (Table 4).

**Table 4:** **Discriminative properties with respect to prevalent vertebral fractures**

OPAQ Scales	Mean Number of Prevalent Vertebral Fractures					p-value			
	0 (n =436)	1 (n =563)	2 (n =257)	3 (n =102)	≥ 4 (n =116)	Linear Trend^a^	0 vs. ≥1^b^	0-1 vs. ≥2	0 vs. ≥2
Walking/bending	87.78	85.12	83.68	78.37	72.82	<0.0001***	0.0054*	<0.0001***	<0.0001***
Standing/sitting	85.20	79.12	76.78	70.87	63.15	<0.0001***	<0.0001***	<0.0001***	<0.0001***
Dressing/reaching	93.60	92.93	91.93	87.75	85.78	<0.0001***	0.4923	0.0030*	0.0557
Household/self-care	94.63	92.28	89.84	86.94	84.48	<0.0001***	0.0076*	<0.0001***	<.00001***
Transfers	93.55	90.15	90.60	82.84	83.03	<0.0001***	0.0030*	0.0012*	0.0002**
Usual work	93.92	91.29	89.26	86.39	80.22	<0.0001***	0.0078*	<0.0001***	0.0002**
Fear of falls	76.51	72.36	70.38	64.47	61.47	<0.0001***	0.0030*	<0.0001***	<.00001***
Level of tension	65.60	68.63	66.94	64.33	66.38	0.3084	0.0604	0.1486	0.8017
Body image	68.95	64.51	63.48	59.42	54.89	<0.0001***	0.0005**	0.0006**	0.0003**
Independence	86.41	83.26	81.20	77.02	74.78	<0.0001***	0.0026*	<0.0001***	<0.0001***
Back pain	78.92	71.97	69.72	61.60	56.61	<0.0001***	<0.0001***	<0.0001***	<0.0001***
Fatigue	63.62	62.34	61.33	56.19	58.26	0.0005**	0.1631	0.0054*	0.0141*
Social activity	39.69	41.12	38.28	39.71	41.59	0.0060*	0.5474	0.0030*	0.0468*
Support, family and friends	83.58	85.51	84.55	89.09	85.67	0.4562	0.2150	0.6822	0.4006

Abbreviations: n = number; OPAQ = Osteoporosis Assessment Questionnaire; vs. = versus.

*p <0.05; **p <0.001; ***p <0.0001.

^a^p-values for linear trend based on analysis of covariance model controlling for country of origin, age, body mass index (BMI), years since menopause, smoking status (yes vs. no), alcohol consumption (yes vs. no), and number of preexisting conditions.

^b^p-values based on analysis of covariance model controlling for country of origin, age, body mass index (BMI), years since menopause, smoking status (yes vs. no), alcohol consumption (yes vs. no), and number of preexisting conditions.

Table 5 provides results related to discriminant validity, against specific known groups. For presence of prevalent osteoporotic nonvertebral fracture (0 vs. ≥1), 3 out of the 14 domains reached statistical significance (household/self-care, transfers, and fear of falls). When 0–1 versus >1 osteoporotic nonvertebral fractures were compared, 7 of the 14 domains reached statistical significance (walking/bending, standing/sitting, household/self-care, transfers, fear of falls, back pain, and fatigue), and these 7 OPAQ 2.0 domains were able to detect a linear trend. For femoral neck BMD T-scores (≥ −2.5 vs. < −2.5), 6 domains (walking/bending, dressing/reaching, household/self-care, usual work, fear of falls, and independence) were statistically significant (p <0.05 or p <0.001). Overall, older patients had lower HRQoL, 7 domains (household/self-care, fear of falls, level of tension, independence, fatigue, social activity, and support of family and friends) for <65, 65 ≤ age ≤70, and >70 detected a linear trend, while 4 domains (walking/bending, standing/sitting, household/self-care, and transfers) reached statistical significance (Table 6).

**Table 5:** **Discriminative properties with prevalent osteoporotic nonvertebral fracture and femoral neck BMD**

OPAQ Scales	Osteoporotic Nonvertebral Fracture							Baseline Femoral Neck BMD T-score		
	0 (n =680)	≥ 1 (n =797)	p-value 0 vs. ≥1	0-1 (n =1096)	> 1 (n =381)	p-value 0–1 vs. >1	Linear Trend^a^	≥ −2.5 (n =884)	< −2.5 (n =585)	p-value^b^
Walking/bending	85.66	83.03	0.0645	85.29	81.22	0.0187*	0.0109*	84.90	83.23	0.0009**
Standing/sitting	80.10	77.53	0.1900	79.95	75.18	0.0363*	0.0219*	79.32	77.79	0.0591
Dressing/reaching	92.66	91.52	0.7054	92.15	91.75	0.4842	0.9948	92.69	91.07	0.0292*
Household/self-care	93.33	90.08	0.0025*	92.40	89.24	0.0422*	0.0076*	92.73	89.83	0.0014*
Transfers	92.23	88.43	0.0038*	91.56	86.20	0.0004**	0.0011*	89.96	90.51	0.4672
Usual work	91.65	89.55	0.2040	91.28	88.32	0.1198	0.1692	91.64	88.81	0.0030*
Fear of falls	74.87	69.28	<0.0001***	73.89	66.03	<0.0001***	<0.0001***	73.02	70.09	0.0061*
Level of tension	67.00	66.91	0.8235	67.17	66.34	0.6539	0.8418	66.90	67.04	0.9125
Body image	65.43	63.82	0.4723	65.46	62.00	0.1254	0.2605	64.41	64.79	0.8934
Independence	83.91	81.76	0.2340	83.44	80.76	0.2742	0.0605	84.01	80.83	0.0040*
Back pain	73.55	70.17	0.0544	73.26	67.31	0.0065*	0.0045*	72.25	70.92	0.1295
Fatigue	62.89	60.91	0.1529	62.71	59.28	0.0291*	0.0343*	62.04	61.49	0.2692
Social activity	39.73	40.54	0.9755	39.90	40.95	0.9519	0.5441	40.43	39.78	0.1497
Support, family and friends	84.49	85.55	0.5963	84.85	85.66	0.6097	0.4871	84.81	85.44	0.8636

Abbreviations: BMD = bone mineral density; n = number; OPAQ = Osteoporosis Assessment Questionnaire; vs. = versus.

^a,b^p-values based on analysis of covariance model controlling for country of origin, age, body mass index (BMI), years since menopause, smoking status (yes vs. no), alcohol consumption (yes vs. no), and number of preexisting conditions.

*p < 0.05; **p < 0.001; ***p < 0.0001.

**Table 6:** **Discriminative properties with respect to age**

	Mean Age			p-value	
OPAQ Scales	< 65 (n =427)	65 ≤ x ≤70 (n =378)	> 70 (n =672)	Linear Trend^a^	p-value^b^
Walking/bending	85.40	85.27	82.92	0.3982	0.0285*
Standing/sitting	80.91	79.48	76.89	0.9784	0.0393*
Dressing/reaching	93.81	92.58	90.64	0.1771	0.2491
Household/self-care	94.00	92.99	89.24	0.0285*	0.0095*
Transfers	91.29	90.52	89.28	0.4317	0.0111*
Usual work	92.73	91.47	88.58	0.7529	0.4834
Fear of falls	77.54	72.13	68.08	<0.0001***	0.3762
Level of tension	63.95	67.63	68.49	<0.0001***	0.6880
Body image	65.74	66.89	62.50	0.6496	0.0966
Independence	85.59	83.56	80.49	0.0029*	0.2028
Back pain	74.69	71.91	69.73	0.7442	0.0922
Fatigue	61.35	61.90	62.07	0.0016*	0.9252
Social activity	39.44	39.79	40.85	0.0034*	0.6037
Support, family and friends	82.57	85.42	86.44	<0.0001***	0.6565

Abbreviations: n = number; OPAQ = Osteoporosis Assessment Questionnaire.

*p < 0.05; **p < 0.001; ***p < 0.0001.

^a^p-values for linear trend based on analysis of covariance model controlling for country of origin, age, body mass index (BMI), years since menopause, smoking status (yes vs. no), alcohol consumption (yes vs. no), and number of preexisting conditions.

^b^p-values based on analysis of covariance model controlling for country of origin, age, body mass index (BMI), years since menopause, smoking status (yes vs. no), alcohol consumption (yes vs. no), and number of preexisting conditions.

Table 7 provides results related to responsiveness to clinical changes (i.e. incident vertebral fractures). Women with ≥1 incident vertebral fracture generally had a greater loss in HRQoL (excluding social activity and support of family and friends) from baseline to study endpoint, compared with women without incident vertebral fractures. There were statistically significant differences in the mean change from baseline to study endpoint, between the 2 groups, in walking/bending (p <0.05), standing/sitting (p <0.05), household/self-care (p <0.001), transfers (p <0.05), usual work (p <0.05), level of tension (p <0.05), independence (p <0.05), and back pain (p <0.001); however, 6 domains (dressing/reaching, fear of falls, body image, fatigue, social activity, and support of family and friends) did not reach statistical significant differences.

**Table 7:** **Association between incident vertebral fractures and mean change in OPAQ at endpoint**

OPAQ Scales	0 Incident Vertebral Fracture (n =918)	≥ 1 Incident Vertebral Fracture (n =155)	p-value^a^
Walking/bending	−1.33 ± 14.49	−5.22 ± 20.92	0.0071*
Standing/sitting	0.47 ± 17.40	−3.30 ± 22.35	0.0165*
Dressing/reaching	−1.19 ± 16.73	−3.76 ± 21.91	0.1368
Household/self-care	−0.90 ± 14.47	−5.42 ± 19.28	0.0012*
Transfers	−0.29 ± 15.53	−3.45 ± 26.57	0.0489*
Usual work	−1.12 ± 17.01	−6.09 ± 25.73	0.0011*
Fear of falls	−3.00 ± 15.72	−3.90 ± 19.23	0.4339
Level of tension	0.96 ± 14.86	−1.78 ± 14.23	0.0449*
Body image	−0.51 ± 22.01	−1.51 ± 25.67	0.8436
Independence	−0.30 ± 14.96	−4.49 ± 19.72	0.0032*
Back pain	0.71 ± 18.76	−5.60 ± 25.46	0.0004**
Fatigue	1.11 ± 16.13	−0.33 ± 14.95	0.1862
Social activity	3.68 ± 19.84	3.19 ± 19.53	0.9628
Support, family and friends	−0.01 ± 18.04	0.90 ± 19.46	0.4000

Abbreviations: n = number; OPAQ = Osteoporosis Assessment Questionnaire.

Results are shown as the mean ± standard deviation change from baseline to study endpoint.

^a^ p-values based on analysis of covariance model controlling for country of origin, age, body mass index (BMI), years since menopause, smoking status (yes vs. no), alcohol consumption (yes vs. no), and number of preexisting conditions.

*p < 0.05; **p < 0.001.

## Discussion

The study assessed the reliability, construct validity, and responsiveness of the OPAQ 2.0 in a subset of women from the MORE study population. The internal consistency reliability was acceptable for the majority of the OPAQ 2.0 domains, and construct validity was demonstrated by using convergent and discriminant analyses. Domains with good psychometric properties included walking/bending, standing/sitting, household/self-care, transfers, usual work, fear of falls, independence, and back pain. Domains with borderline psychometric properties included fatigue and social activity. Domains lacking good psychometric properties included dressing/reaching, level of tension, body image, and support of family and friends.

Previous versions of the OPAQ were tested in small populations (N =40), and the results suggested that OPAQ is a reliable, consistent, and valid instrument capable of distinguishing hierarchy of functional loss in disease states in osteoporosis [9]. This study found similar results in a larger multicenter international population of postmenopausal women. Payers are increasingly insisting on economic evaluations, such as cost-effectiveness analyses and cost-utility analyses (which rely on health utility assessment) for new treatments to support reimbursement decisions. In studies in which utility scores are not collected, given the high correlation between HRQoL and utility, one would expect that improvement in HRQoL may be reflected in improvements in health utility, which could aid in cost-effectiveness assessment.

The HRQoL measures are more commonly included as an outcome measure alongside BMD measurements and the assessment of vertebral fracture incidence [14, 17]. Because sudden changes in HRQoL may reflect changes in the progression of disease (e.g. subsequent fractures) and given that osteoporosis is a silent disease, (e.g. vertebral fractures or deformities may go undiagnosed and risk for fractures can still occur with a normal BMD [25]), detecting the worsening of disease early is important to manage treatment success and to avoid further consequences (e.g. hip fracture).

All but 2 OPAQ domains—level of tension and support of family and friends—were able to discriminate between patients with or without prevalent vertebral fractures and to detect a reduced HRQoL with increased number of vertebral fractures. Similar results were previously reported with the OPAQ, the Quality-of-life questionnaire of the European Foundation for Osteoporosis (QUALEFFO), and the geriatric depression scale [6, 14, 15]. However, results differed in that no significant associations were seen in the social interaction domains in OPAQ 2.0, which could be due to differences between the OPAQ 2.0 and QUALEFFO.

For osteoporotic nonvertebral fractures, OPAQ 2.0 was better able to detect a reduced HRQoL and to discriminate between patients with 0 to 1 versus >1 nonvertebral fractures—where 7 of 14 domains were statistically significant and also were associated with a worsening trend with increased number of vertebral fractures—than for patients with 0 versus ≥1 fracture—where only 3 domains (household/self-care, transfers, and fear of falls) reached statistical significance. Most of the domains that did not show statistical significance are not reflective of one’s individual physical ability; they are mostly influenced by the support of external parties. Several studies have shown an adverse impact in HRQoL (mostly in the physical function, emotional status, and symptoms dimensions) with nonvertebral fractures; however, different instruments were used to measure HRQoL, and our study uniquely assessed vertebral and nonvertebral fractures separately, whereas other studies did not [22, 26–28].

For femoral neck BMD T-scores, most domains did not discriminate well. This result could be because a BMD of ≤ −2.5 may not fully represent the impact of severe osteoporosis. Patients with BMD measurements above the osteoporosis threshold of −2.5 still report fractures. In the National Osteoporosis Risk Assessment study, postmenopausal women with BMD ≤ −2.5 had the highest rate of fractures (18% of osteoporotic fractures and 26% of hip fractures); however, approximately 23% of women had a BMD ≤ −2.0 or ≤ −1.5 with 1 or more clinical risk factors, and though fracture rates were lower, 45% of osteoporotic fractures and 53% of hip fractures occurred in these women [29]. Most people do not know their BMD is ≤2.5 until after a BMD test is performed; however, most quality of life domain scores begin to separate quickly between low BMD groups, if a fracture has occurred.

Overall, older patients had lower HRQoL. Fractures are often undiagnosed, and 1 study revealed that osteoporosis or vertebral fracture was diagnosed in <2% of white women ≥60 years old, but the prevalence of these fractures has been found to be 20% to 30% in these women [30]. Osteoporotic fractures generally affect older patients. Vertebral, hip, and wrist fractures are primary causes for morbidity in patients with osteoporosis, and these fractures can lead to acute pain and loss of function [4, 31–34]. These fractures, in turn, can lead to lower quality of life, so these results are not surprising; however, decreasing HRQoL in older patients could also be explained by additional chronic illnesses, comorbidities, and a number of other factors.

Women with ≥1 incident vertebral fracture generally had a greater loss in HRQoL from baseline to study endpoint, compared with women without incident vertebral fractures, which is consistent with previous research [6, 26, 27].

There are some important limitations that should be considered when interpreting these findings. This study was a post hoc retrospective analysis. Although the study used data from the MORE study—which was a well-controlled randomized clinical trial with a large, heterogeneous osteoporosis patient population and included multiple HRQoL instruments (including generic, disease-specific, and preference-based instruments)—the trial was conducted in the 1990s and some results may not be as generalizable today. We also were unable to conduct a time to event analysis on HRQoL scores between baseline and the time of vertebral fracture due to the timing of vertebral fracture assessments (baseline, and months 12, 24 and 36).

This study assessed the psychometric properties of the OPAQ at the domain level, whereas previous work focused attention on the dimension level [6, 10, 14]. Results from the current study are consistent with previous research; however, domain-level results have not been disclosed in the past, and for further development and validation of OPAQ and other osteoporosis-specific instruments, domain-level results provide useful information.

The OPAQ 2.0 is a validated, self-administered HRQoL instrument. The OPAQ 2.0 was developed to capture broader dimensions of HRQoL for patients with osteoporosis in clinical trials; however, the length of the original version of OPAQ 2.0 (67 questions) and the resulting respondent time burden could pose a concern for inclusion in clinical trials. Our findings suggest that there are domains that could be reassessed to further refine OPAQ 2.0 to develop a shorter version that has the best psychometric properties for use in clinical trials and routine patient care that follow recent United States Food and Drug Administration guidelines.

## Conclusions

Most of the domains in the 67-item OPAQ instrument (version 2.0) have demonstrated reliability, discriminant validity, and responsiveness. Despite these robust findings, there is a need in clinical trial research to limit the number of items to as few as possible and to minimize patient responder burden. The findings provided here suggest that there are several domains that do not show these criteria and that may need to be reassessed and removed for a potentially shorter and validated version of OPAQ.

## Electronic supplementary material

Additional file 1:The Osteoporosis Assessment Questionnaire (OPAQ™ Version 2.0): osteoporosis treatment study for raloxifene.(PDF 37 KB)

Additional file 2:Scoring algorithms of OPAQ scales and dimensions.(PDF 21 KB)

Below are the links to the authors’ original submitted files for images.

Authors’ original file for figure 1

## Abbreviations

BMD: Bone mineral density
HRQoL: Health-related quality of life
HUI: Health Utilities Index
MORE: Multiple Outcomes of Raloxifene Evaluation
NHP: Nottingham Health Profile
OPAQ: Osteoporosis Assessment Questionnaire
OPAQ 2.0: OPAQ instrument version 2.0
PRO: Patient-reported outcomes
QUALEFFO: Quality-of-life questionnaire of the European Foundation for Osteoporosis.
