# Supplementary material for: Psychometric properties of the osteoporosis assessment questionnaire (OPAQ) 2.0: results from the multiple outcomes of raloxifene evaluation (MORE) study
Source: BMC Musculoskelet Disord. 2014 Nov 17;15:374. doi: 10.1186/1471-2474-15-374 (PMC4246554; doi:10.1186/1471-2474-15-374)
Supplement: Supplementary file 1 — Additional file 1:The Osteoporosis Assessment Questionnaire (OPAQ™ Version 2.0): osteoporosis treatment study for raloxifene.(PDF 37 KB) [file 12891_2014_2311_MOESM1_ESM.pdf]

THE OSTEOPOROSIS ASSESSMENT QUESTIONNAIRE
(OPAQ™ Version 2.0)

Osteoporosis Treatment Study For Raloxifene

Patient Number __________

Site Number __________

Investigator Number __________

Visit Number __________

North America/Australia/New Zealand

Please answer the following questions about your health. Most questions ask about your health during the past two weeks. There are no right or wrong answers to the questions and most can be answered with a simple “3” or “5”. It is very important that you answer every question.

1. Would you please circle the number that best indicates how you would rate your quality of life related to health or illness at this time.

☺	10	9	8	7	6	5	4	3	2	1	0	☹

Best Possible Quality of Life — Worst Possible Quality of Life

2. Would you please circle the number that best indicates how you would rate your overall quality of life at this time.

☺	10	9	8	7	6	5	4	3	2	1	0	☹

Best Possible Quality of Life — Worst Possible Quality of Life

3. Compared to one year ago, how has your overall quality of life changed, if any?

_____ a. ☺☺☺ The best it has ever been
_____ b. ☺☺ Much better
_____ c. ☺ Somewhat better
_____ d. 😐 No change; it is the same as one year ago
_____ e. ☹ Somewhat worse
_____ f. ☹☹ Much worse
_____ g. ☹☹☹ The worst it has ever been

4. Compared to one year ago, how has your health- or illness-related quality of life changed, if any?

_____ a. ☺☺☺ The best it has ever been
_____ b. ☺☺ Much better
_____ c. ☺ Somewhat better
_____ d. 😐 No change; it is the same as one year ago
_____ e. ☹ Somewhat worse
_____ f. ☹☹ Much worse
_____ g. ☹☹☹ The worst it has ever been

5. If your overall quality of life has changed in the past year, why has it changed? Check all answers that apply.

_____ a. Financial changes
_____ b. Changes in my physical health

______ c. Changes in my emotional health
______ d. Changes in my social life
______ e. Changes in my family or friends
______ f. I do not know
______ g. No change in my quality of life

6. Please check all answers that describe your current living situation.

_____ a. I live alone in a house or apartment
_____ b. I live with a spouse or partner in a house or apartment
_____ c. I have someone who helps me with heavy housework
_____ d. I have someone who helps me with most of my housework
_____ e. I have someone who helps me when I do activities outside the home
_____ f. I have a full-time aide who helps me during the day
_____ g. I live in the house of a family member or friend
_____ h. I live in a board-and-care facility or other group facility
_____ i. I live in a nursing home

The next 7 questions refer to WALKING AND BENDING.

DURING THE PAST TWO WEEKS:

7. How often were you able to do daily shopping or errands?

_____ a. All Days
_____ b. Most Days
_____ c. Some Days
_____ d. Few Days
_____ e. No Days

8. How often were you in a bed or chair for most of the day?

_____ a. All Days
_____ b. Most Days
_____ c. Some Days
_____ d. Few Days
_____ e. No Days

9. How often were you able to do sports and games that you would like to do?

_____ a. All Days
_____ b. Most Days
_____ c. Some Days
_____ d. Few Days
_____ e. No Days

10. How often were you able to walk as much as you needed to do?

_____ a. All Days
_____ b. Most Days
_____ c. Some Days

_____ d. Few Days
_____ e. No Days

11. How often did you have trouble bending, lifting or stooping?

_____ a. All Days
_____ b. Most Days
_____ c. Some Days
_____ d. Few Days
_____ e. No Days

12. How often did you have trouble either walking one block or climbing one flight of stairs?

_____ a. All Days
_____ b. Most Days
_____ c. Some Days
_____ d. Few Days
_____ e. No Days

13. How often did you need to use a cane, crutches, walker, or companion while walking?

_____ a. All Days
_____ b. Most Days
_____ c. Some Days
_____ d. Few Days
_____ e. No Days

The next 3 questions refer to STANDING AND SITTING.

DURING THE PAST TWO WEEKS:

14. How often did you have trouble standing as much as you needed to do?

_____ a. All Days
_____ b. Most Days
_____ c. Some Days
_____ d. Few Days
_____ e. No Days

15. How often did you have trouble sitting as much as you needed to do?

_____ a. All Days
_____ b. Most Days
_____ c. Some Days
_____ d. Few Days
_____ e. No Days

16. How often did you feel that your back tired easily?

_____ a. All Days
_____ b. Most Days
_____ c. Some Days
_____ d. Few Days
_____ e. No Days

The next 3 questions refer to DRESSING AND REACHING TASKS.

DURING THE PAST TWO WEEKS:

17. Could you easily put on or take off a pair of stockings and/or underwear?

_____ a. All Days
_____ b. Most Days
_____ c. Some Days
_____ d. Few Days
_____ e. No Days

18. Could you easily comb, brush, or style your hair?

_____ a. All Days
_____ b. Most Days
_____ c. Some Days
_____ d. Few Days
_____ e. No Days

19. Could you easily reach shelves that were above your head?

_____ a. All Days
_____ b. Most Days
_____ c. Some Days
_____ d. Few Days
_____ e. No Days

The next 4 questions refer to HOUSEHOLD AND SELF-CARE TASKS.

DURING THE PAST TWO WEEKS:

20. Have you had to change the way you bathe yourself?

_____ a. Always
_____ b. Very Often
_____ c. Sometimes
_____ d. Almost Never
_____ e. Never

21. Have you had to change the types of clothes you wear because of difficulty in dressing?

_____ a. Always
_____ b. Very Often
_____ c. Sometimes
_____ d. Almost Never
_____ e. Never

22. How often were you able to do light housework such as cooking without help?

_____ a. Always
_____ b. Very Often
_____ c. Sometimes
_____ d. Almost Never
_____ e. Never

23. How often were you able to do heavy housework such as vacuuming without help?

_____ a. Always
_____ b. Very Often
_____ c. Sometimes
_____ d. Almost Never
_____ e. Never

The next 4 questions refer to TRANSFERS (Getting up and down).

DURING THE PAST TWO WEEKS:

24. How often did you have trouble getting in or out of bed?

_____ a. All Days
_____ b. Most Days
_____ c. Some Days
_____ d. Few Days
_____ e. No Days

25. How often did you have trouble getting in or out of a chair?

_____ a. All Days
_____ b. Most Days
_____ c. Some Days
_____ d. Few Days
_____ e. No Days

26. How often did you have trouble getting on or off the toilet?

_____ a. All Days
_____ b. Most Days
_____ c. Some Days
_____ d. Few Days
_____ e. No Days

27. How often did you have trouble getting in and out of cars or public transportation?

_____ a. All Days
_____ b. Most Days
_____ c. Some Days
_____ d. Few Days
_____ e. No Days

The next 5 questions refer to FALLS.

DURING THE PAST TWO WEEKS:

28. How often were you afraid that you would fall?

_____ a. Always
_____ b. Very Often
_____ c. Sometimes

_____ d. Almost Never
_____ e. Never

29. How often were you afraid that you would accidentally break or fracture a bone?

_____ a. Always
_____ b. Very Often
_____ c. Sometimes
_____ d. Almost Never
_____ e. Never

30. How often did you feel that you were losing your balance?

_____ a. Always
_____ b. Very Often
_____ c. Sometimes
_____ d. Almost Never
_____ e. Never

31. How often did you use a handrail or other support when walking up or down stairs?

_____ a. Always
_____ b. Very Often
_____ c. Sometimes
_____ d. Almost Never
_____ e. Never

32. How often did fear of falling keep you from doing what you wanted to do?

_____ a. Always
_____ b. Very Often
_____ c. Sometimes
_____ d. Almost Never
_____ e. Never

The next 3 questions refer to SOCIAL ACTIVITY.

DURING THE PAST TWO WEEKS:

33. How often did you have friends or relatives over to your house?

_____ a. All Days
_____ b. Most Days
_____ c. Some Days
_____ d. Few Days
_____ e. No Days

34. How often did you visit friends or relatives at their homes?

_____ a. All Days
_____ b. Most Days
_____ c. Some Days
_____ d. Few Days
_____ e. No Days

35. How often were you able to go to church, meetings, movies or other outside activities?

_____ a. All Days
_____ b. Most Days
_____ c. Some Days
_____ d. Few Days
_____ e. No Days

The next 2 questions refer to SUPPORT FROM FAMILY AND FRIENDS.

DURING THE PAST TWO WEEKS:

36. Did you feel that your family or friends would be around if you needed assistance?

_____ a. Always
_____ b. Very Often
_____ c. Sometimes
_____ d. Almost Never
_____ e. Never

37. Did you feel that your family or friends were sensitive to your personal needs?

_____ a. Always
_____ b. Very Often
_____ c. Sometimes
_____ d. Almost Never
_____ e. Never

The next 4 questions refer to BACK ACHE AND PAIN.

DURING THE PAST TWO WEEKS:

38. How often did you have any back ache or pain?

_____ a. All Days
_____ b. Most Days
_____ c. Some Days
_____ d. Few Days
_____ e. No Days

39. How would you describe the back ache or pain you usually had?

_____ a. Severe
_____ b. Moderate
_____ c. Mild
_____ d. Very Mild
_____ e. None, I had no back pain

40. How often did your back feel stiff for more than one hour from the time you woke up?

_____ a. All Days
_____ b. Most Days

_____ c. Some Days
_____ d. Few Days
_____ e. No Days

41. How often did back ache or pain keep you from doing what you wanted to do?

_____ a. All Days
_____ b. Most Days
_____ c. Some Days
_____ d. Few Days
_____ e. No Days

The next 2 questions refer to FATIGUE.

DURING THE PAST TWO WEEKS:

42. How often did you feel well rested in the morning?

_____ a. Always
_____ b. Very Often
_____ c. Sometimes
_____ d. Almost Never
_____ e. Never

43. How often did you feel tired during the day?

_____ a. Always
_____ b. Very Often
_____ c. Sometimes
_____ d. Almost Never
_____ e. Never

The next question refers to your USUAL WORK.

DURING THE PAST TWO WEEKS:

44. How often were you able to do your usual daily work, either at home, as a volunteer, at school, or at a paid job?

_____ a. Always
_____ b. Very Often
_____ c. Sometimes
_____ d. Almost Never
_____ e. Never

The next 5 questions refer to LEVEL OF TENSION

DURING THE PAST TWO WEEKS:

45. How often have you felt tense or high strung?

_____ a. Always
_____ b. Very Often
_____ c. Sometimes
_____ d. Almost Never
_____ e. Never

46. How often have you been bothered by nervousness or your nerves?

_____ a. Always
_____ b. Very Often
_____ c. Sometimes
_____ d. Almost Never
_____ e. Never

47. How often have you felt stressed by your daily life?

_____ a. Always
_____ b. Very Often
_____ c. Sometimes
_____ d. Almost Never
_____ e. Never

48. How often have you felt relaxed and free of tension?

_____ a. Always
_____ b. Very Often
_____ c. Sometimes
_____ d. Almost Never
_____ e. Never

49. How often have you felt calm and peaceful?

_____ a. Always
_____ b. Very Often
_____ c. Sometimes
_____ d. Almost Never
_____ e. Never

The next 3 questions refer to BODY IMAGE.

DURING THE PAST TWO WEEKS:

50. How often were you aware of changes in your body when trying on clothes?

_____ a. Always
_____ b. Very Often
_____ c. Sometimes
_____ d. Almost Never
_____ e. Never

51. How often were you bothered by the way your back looks?

_____ a. Always
_____ b. Very Often
_____ c. Sometimes
_____ d. Almost Never
_____ e. Never

52. How often were you concerned by changes in the way your body looks?

______ a. Always
______ b. Very Often
______ c. Sometimes
______ d. Almost Never
______ e. Never

The next 3 questions refer to INDEPENDENCE.

DURING THE PAST TWO WEEKS:

53. How often did you feel confident you could live on your own without assistance?

_____ a. Always
_____ b. Very Often
_____ c. Sometimes
_____ d. Almost Never
_____ e. Never

54. How often did you have to rely on others for assistance in daily activities?

_____ a. Always
_____ b. Very Often
_____ c. Sometimes
_____ d. Almost Never
_____ e. Never

55. How often were you worried that you might not be able to take care of yourself in the future?

_____ a. Always
_____ b. Very Often
_____ c. Sometimes
_____ d. Almost Never
_____ e. Never

The next 12 questions ask you to rate the importance of different daily activities to you.

IMPORTANCE OF DAILY ACTIVITIES:

56. How important to you is doing your daily shopping or errands?

_____ a. Not Important
_____ b. Somewhat Important
_____ c. Very Important
_____ d. Extremely Important

57. How important to you is being able to do sports and games that you would like to do?

_____ a. Not Important
_____ b. Somewhat Important
_____ c. Very Important

_____ d. Extremely Important

58. How important to you is being able to do heavy housework such as vacuuming?

_____ a. Not Important
_____ b. Somewhat Important
_____ c. Very Important
_____ d. Extremely Important

59. How important to you is being able to do light housework such as cooking?

_____ a. Not Important
_____ b. Somewhat Important
_____ c. Very Important
_____ d. Extremely Important

60. How important to you is being able to do your usual daily work, either at home, as a volunteer, at school, or at a paid job?

_____ a. Not Important
_____ b. Somewhat Important
_____ c. Very Important
_____ d. Extremely Important

61. How important to you is spending time with your friends and family?

_____ a. Not Important
_____ b. Somewhat Important
_____ c. Very Important
_____ d. Extremely Important

62. How important to you is walking as much as you need to do?

_____ a. Not Important
_____ b. Somewhat Important
_____ c. Very Important
_____ d. Extremely Important

63. How important to you is going to church, meetings, movies, or other outside activities?

_____ a. Not Important
_____ b. Somewhat Important
_____ c. Very Important
_____ d. Extremely Important

64. How important to you is getting out of your bed or chair for most of the day?

_____ a. Not Important
_____ b. Somewhat Important
_____ c. Very Important
_____ d. Extremely Important

65. How important to you is feeling free of back pain?

_____ a. Not Important
_____ b. Somewhat Important
_____ c. Very Important
_____ d. Extremely Important

66. How important to you is your emotional health (feeling anxious, depressed, or stressed out)?

_____ a. Not Important
_____ b. Somewhat Important
_____ c. Very Important
_____ d. Extremely Important

67. How important to you is being independent?

_____ a. Not Important
_____ b. Somewhat Important
_____ c. Very Important
_____ d. Extremely Important

Thank you very much for your time.
