# Supplementary material for: Psychometric properties of the osteoporosis assessment questionnaire (OPAQ) 2.0: results from the multiple outcomes of raloxifene evaluation (MORE) study
Source: BMC Musculoskelet Disord. 2014 Nov 17;15:374. doi: 10.1186/1471-2474-15-374 (PMC4246554; doi:10.1186/1471-2474-15-374)
Supplement: Supplementary file 2 — Additional file 2:Scoring algorithms of OPAQ scales and dimensions.(PDF 21 KB) [file 12891_2014_2311_MOESM2_ESM.pdf]

Scoring algorithms of OPAQ scales and dimensions

OPAQ Scale	Number of Questions	Question	Recoding
Walking/Bending	7	7 - 13	7, 9, 10
Standing/Sitting	3	14 - 16	None
Dressing/Reaching	3	17 - 19	17, 18, 19
Household/Self-Care	4	20 - 23	22, 23
Transfers	4	24 - 27	None
Fear of Falls	5	28 - 32	None
Social Activity	2	33 - 34	33, 34
Support, Family and Friend	2	36 - 37	36, 37
Back Pain	4	38 - 41	None
Fatigue	2	42 - 43	42
Usual Work	1	44	44
Level of Tension	5	45 - 49	48, 49
Body Image	3	50 - 52	None
Independence	3	53 - 55	53

Abbreviation: OPAQ= Osteoporosis Patient Assessment Questionnaire.
