# Supplementary material for: Psychometric properties of the osteoporosis assessment questionnaire (OPAQ) 2.0: results from the multiple outcomes of raloxifene evaluation (MORE) study
Source: BMC Musculoskelet Disord. 2014 Nov 17;15:374. doi: 10.1186/1471-2474-15-374 (PMC4246554; doi:10.1186/1471-2474-15-374)
Supplement: Supplementary file 3 — Authors’ original file for figure 1 [file 12891_2014_2311_MOESM3_ESM.pdf]

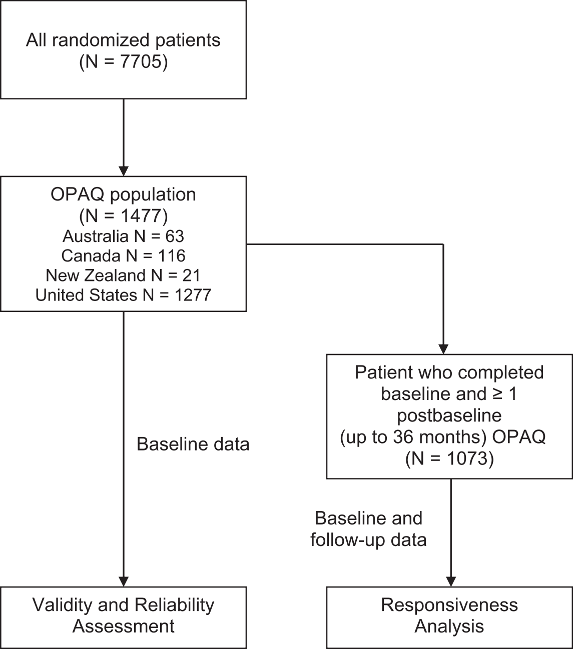

All randomized patients
(N = 7705)
OPAQ population
(N = 1477)
Australia N = 63
Canada N = 116
New Zealand N = 21
United States N = 1277
Patient who completed
baseline and ≥ 1
postbaseline
(up to 36 months) OPAQ
(N = 1073)
Baseline data
Baseline and
follow-up data
Validity and Reliability
Assessment
Responsiveness
Analysis
